# Validation of Wireless Sensors for Psychophysiological Studies

**DOI:** 10.3390/s19224824

**Published:** 2019-11-06

**Authors:** Pedro Silva Moreira, Pedro Chaves, Ruben Dias, Nuno Dias, Pedro R Almeida

**Affiliations:** 1 Life and Health Sciences Research Institute (ICVS), School of Medicine, University of Minho, 4710-057 Braga, Portugal; 2 ICVS/3B’s, PT Government Associate Laboratory, 4710-057 Braga/Guimarães, Portugal; 3 MindProber Labs, 4450-102 Porto, Portugal; pedro.chaves@mindproberlabs.com (P.C.); ruben.dias@mindproberlabs.com (R.D.); nuno.dias@mindproberlabs.com (N.D.); pedro.almeida@mindproberlabs.com (P.R.A.); 4 Department of Experimental Biology, Faculty of Medicine, University of Porto, 4200-319 Porto, Portugal; 5 2Ai-Polytechnic Institute of Cávado and Ave, Campus do IPCA, 4750-810 Barcelos, Portugal; 6 School of Criminology, Faculty of Law, University of Porto, 4050-123 Porto, Portugal

**Keywords:** psychophysiology, electrodermal activity, heart rate

## Abstract

James One (MindProber Labs) is a wireless psychophysiological device comprising two sensors: one measuring electrodermal activity (EDA), the other photoplethysmography (PPG). This paper reports the validation of James One’s EDA sensor by comparing its signal against a research grade polygraph. Twenty participants were instructed to perform breathing exercises to elicit the modulation of EDA and heart rate, while the physiological signal was captured simultaneously on James One and a Biopac MP36. The resulting EDA and PPG records collected from both systems were comprehensively compared. Results suggest that James One captures EDA signal with a quality comparable to a research grade equipment, this constituting a reliable means of capturing data while minimizing setup time and intrusiveness.

## 1. Introduction

Psychophysiology is a branch of science directly associated with anatomy, physiology, and psychological processes. It aims to elucidate about the structure and function of interrelated systems in the human body in response to environmental contingencies [1]. Several psychophysiological measures have been used across the state-of-the-art, including central measures (e.g., electroencephalography and functional magnetic resonance imaging) and peripheral measures, encompassing electrodermal, cardiovascular, respiratory, and facial systems. Here, we focus on the electrodermal and cardiac groups of the peripheral psychophysiological measures. In short, electrodermal activity (EDA) corresponds to the electrical conductance of the skin, dependent of variations of sweat secreted by the eccrine sweat glands. The EDA signal has two components: the tonic component (the skin conductance level, SCL) that constitutes the slower background signal oscillations; the phasic component, which correspond to stimulus-related (the skin conductance response, SCR) or stimulus-independent (the non-specific skin conductance response, NS-SCR) fast-changing oscillations of the signal [2]. EDA has been widely studied in the history of psychophysiology in a variety of different contexts, including attention, information processing, emotion, among others. Notably, such measures have been demonstrated to vary with the salience of stimuli [3]. In a similar fashion, the cardiovascular system has been comprehensively characterized across decades of scientific research. Within the cardiovascular system, measures related to the heart rate (HR, i.e., beats per minute) and heart rate variability [HRV, within the time (e.g., inter-beat interval, root-mean square of successive RR interval differences) and frequency domains (e.g., low and high frequencies of heart rate variability)] have been widely explored [4]. The cardiovascular system has been reported to be involved in domains such as self-regulation, fatigue [5], and emotional processing [6].

Altered patterns within these psychophysiological measures have been observed across distinct psychopathological conditions. A growing number of scientific publications characterizes distinct psychiatric conditions with regards to the functioning of the autonomic nervous system (ANS). For instance, depression is characterized by significantly reduced EDA [7]; heightened skin conductance has been reported in posttraumatic stress disorder [8] and during emotion recognition in borderline personality disorder [9]. HRV—which has been consistently associated with top-down self-regulation [10]—has been proposed as a transdiagnostic biomarker for a variety of psychopathological conditions [11]. The abovementioned examples highlight the significance of distinct psychophysiological measures for a variety of mental health conditions.

A common issue with the typical psychophysiological experiments relies on the fact that the most widely used systems are expensive and non-portable, thus requiring participants to be assessed in laboratorial contexts. This characteristic of the standard psychophysiological laboratories has two important implications: First, individuals are not assessed in their natural context (thus, constituting a threat to ecological validity of the assessments); second, it becomes particularly different to constitute large enough sample sizes to detect less pronounced effects. The increasing use of information and communication technologies for health—eHealth (WHO, 2015)—offers a set of challenges and opportunities for the monitoring and interventions on different populations. The use of such strategies has been shown to enable an improved health literacy [12] and constitutes a reliable approach for the promotion of physical activity in elderly individuals [13]. It has also been reported that eHealth interventions are associated with statistically significant improvements on symptoms of anxiety [14], depression [15], or posttraumatic stress disorder [16]. Thus, mental health conditions may benefit from the integration of robust psychophysiological assessments in eHealth approaches. 

In the present work we aimed to address the lack of scalability and ecological validity of standard psychophysiological monitoring by assessing the reliability of a small, portable, low-cost, wireless system as a proper tool for the monitoring of HR and EDA.

## 2. Materials and Methods

### 2.1. Description of the System

#### 2.1.1. Hardware

**Figure 1:**
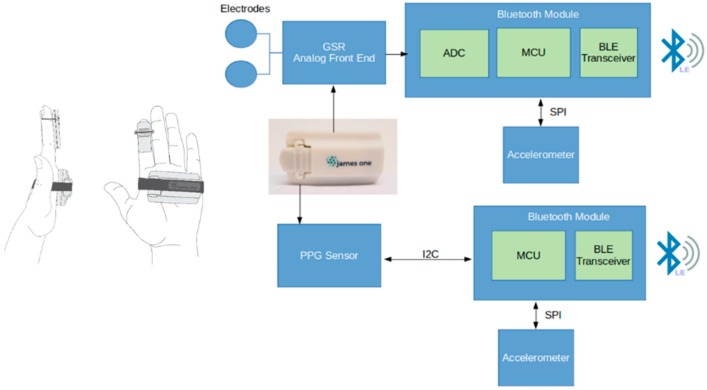
Overview of the James One system.

James One is comprised of two sensors, each one presenting a specific architecture based on the same nRF52832 BLE system-on-chip (SoC) from Nordic Semiconductor. This system-on-chipSoC includes analogue-to-digital converter (ADC), microcontroller unit (MCU), and a Bluetooth low energy (BLE) transceiver. The larger sensor measures electrodermal activity (EDA), through two disposable pre-gelled electrodes connected to an analogue front-end that translates skin conductivity into a voltage signal. The voltage correspondent to the skin conductance is converted by the ADC that is internally available in the MCU. The photoplethysmography (PPG) sensor captures light absorbance (in three different wavelengths through the emission of green, infrared, and red LEDs) by the oxygenated blood that is being pumped by the heart toward the human body periphery. The heart rate in beats per minute is calculated by a proprietary algorithm that runs in the MCU of the PPG sensor. Both EDA and PPG sensors employ a switch on/off mechanism based on the movement detected by the accelerometer and send data at 100 samples/s to any external device with Bluetooth connectivity. An overview of this system is graphically represented on Figure 1. 

Electrodermal activity (EDA) is dependent on variations of sweat secreted by eccrine sweat glands in the hypodermis [17] and measures responses of autonomic nervous system to timely stimuli presented to the user. As presented on the Figure 1, the EDA sensor is applied on the hand palm and the PPG sensor is preferably applied on a fingertip. Both signals from James One were sampled at 100 Hz. The state-of-the-art has demonstrated that this sampling rate enables a reliable estimation of heart rate variability (HRV) metrics [18].

#### 2.1.2. Software

We used an in-house developed software for the real-time visualization and recording of both signals. The API permits the receiving of data frames from the plethysmography PPG and EDA signals. The software was developed using different Python libraries, running on a Raspberry Pi-3 and the Raspbian OS. The API has a graphical user interface which allows to visualize and record the signals in real-time.

#### 2.1.3. Participants

Twenty college students (12 females) with an average age of 26.95 years (SD = 5.62) were recruited for this study. The goals of the experiment were carefully explained to all the participants. Participants were also informed that the participation on the study was volunteer and that they could leave the experiment at any time. None of the participants had history of neurologic and/or psychiatric disorders.

#### 2.1.4. Psychophysiological Recordings

The experiment was conducted in a sound-attenuated room, equipped with a comfortable armchair. To maximize the quality of the signal, the skin of the participants was cleaned to remove excessive sweating. For this experiment, a Biopac MP36 Psychophysiological Monitoring System was used for the collection of psychophysiological measures. EDA was recorded using an SS57LA EDA lead set, which applies a constant 0.5 Volts Direct Current across two disposable Ag-AgCl skin electrodes (placed on the thenar and hypothenar eminences of the left hand) and then measures the current flowing between them. Heart rate was monitored with an TSD200 PPG transducer (attached to the index finger of the left hand), which records the blood volume pulse waveform. Both signals from the Biopac MP36 system were sampled at 500 Hz. The signal was recorded using the Biopac Student Lab system, running on a MacBook Air. The James One sensors were placed on participants’ right hand. All the signals were continuously monitored throughout the experiment.

#### 2.1.5. Experimental Apparatus

The experiment followed a structured fashion across participants. The recording started with a 60 s baseline acquisition, in which participants were instructed to remain calm and still and to focus on a fixation cross that was presented on a 15.6” full-HD display. After the baseline period, a short monotonic sound (0.5 s) was coupled with a visual instruction to breath-in (duration = 5 s), followed by a breath-out instruction (duration = 5 s) and by a resting period of 15 seconds. This sequence (breath-in—breath-out—rest) was repeated five times. The visual instructions were triggered in the data. For the purpose of this investigation, the psychophysiological signals were processed and analyzed offline as described in the following section.

### 2.2. Data Analysis

All the data processing was implemented with Python libraries, including *bioread* (for loading .acq files obtained from the biopac system), *pandas* and *numpy* (for data manipulation and general computing), *scipy* (for mathematical operations related with signal processing), *heartpy* (for the peak detection and calculation of HR metrics), *ledapy* (for the decomposition of the EDA signal), *matplotlib* and *seaborn* (for plotting). 

#### 2.2.1. Comparison of the EDA signal

The similarity between the physiological signals recorded with the different recording systems was analyzed by (1) down-sampling of the Biopac signal from 500 Hz to 100 Hz (to meet the sampling rate of James One recordings); band-pass filtering (low cutoff: 0.03 Hz; high cutoff: 1 Hz) (2) temporal alignment between the signals; (3) computation of the cross-correlation between the signals, which is typically used to compare the similarity between the two time-series. For such statistical procedures, we considered the EDA signal both in raw units (measured in microsiemens, μS) as well as the standardized units (z-Scores). To test the association between different components of the signal, a continuous decomposition analysis (CDA) [19] was performed to decompose the raw signal into tonic and phasic components. After the decomposition of the signals, the number of skin conductance responses (SCRs) was quantified. The resulting number of SCRs were statistically compared between the James One and Biopac devices. 

The time-series of the signals obtained with the different sensors were statistically compared with regards to the tonic and phasic components of the signal. Nevertheless, whereas correlation quantifies the strength of a relationship between two variables are related, it does not imply that there is good agreement between the two methods [20]. Thus, for assessing the agreement between the two signals, Bland–Altman plots were generated. Such graphical representation allows to quantify the variation in the differences between methods [21]. For two methods to agree, it is recommended that 95% of the observations lie within 1.96 standard deviations from the mean difference between the signals.

#### 2.2.2. Comparison of the PPG Signal

After down-sampling the Biopac signal to 100 Hz and aligning the signals from both sensors, the signals were processed using bandpass filtering using a low cutoff of 0.5 Hz and a high cutoff of 3.0 Hz. The peak detection was performed, considering the whole time-series for each subject. The default arguments were used, including: the use of interpolation of implied peak shapes for clipping parts of the signal, a minimum value to fit peaks of 40 bpm and a maximum value to fit peaks of 180 bpm. The average HR was estimated for the whole time-series. On top of this, a sensitivity analysis was implemented to assess the association between signals across different time intervals. Correlation analyses were implemented to test the association between whole time-series heart rate, as well as between temporal measures of heart-rate variability (HRV), including interbeat interval (IBI), standard deviation of RR intervals (SDNN), standard deviation of successive differences (SDSD), root mean square of successive differences (RMSSD), proportion of successive differences above 20 ms (pNN20), proportion of successive differences above 50 ms (pNN50), median absolute deviation of RR intervals (MAD). In addition, the following HRV metrics were statistically compared for the frequency domain: low-frequency HRV (LF-HRV, range: 0.05–0.15 Hz), high-frequency HRV (HF-HRV, range: 0.15–0.5 Hz), as well as the ration between high-frequency and low-frequency HRV (HF/LF HRV). Furthermore, breathing rate (BR) was also estimated from the PPG signal, as there has been shown that there is a relationship between R-R intervals and breathing rate. We can also exploit this relationship to extract breathing rate from a segment of heart rate data. Fisher r-to-z transformation was used to compute the average of correlation coefficients.

## 3. Results

### 3.1. Electrodermal Activity Raw, Tonic, and Phasic Components

**Figure 2:**
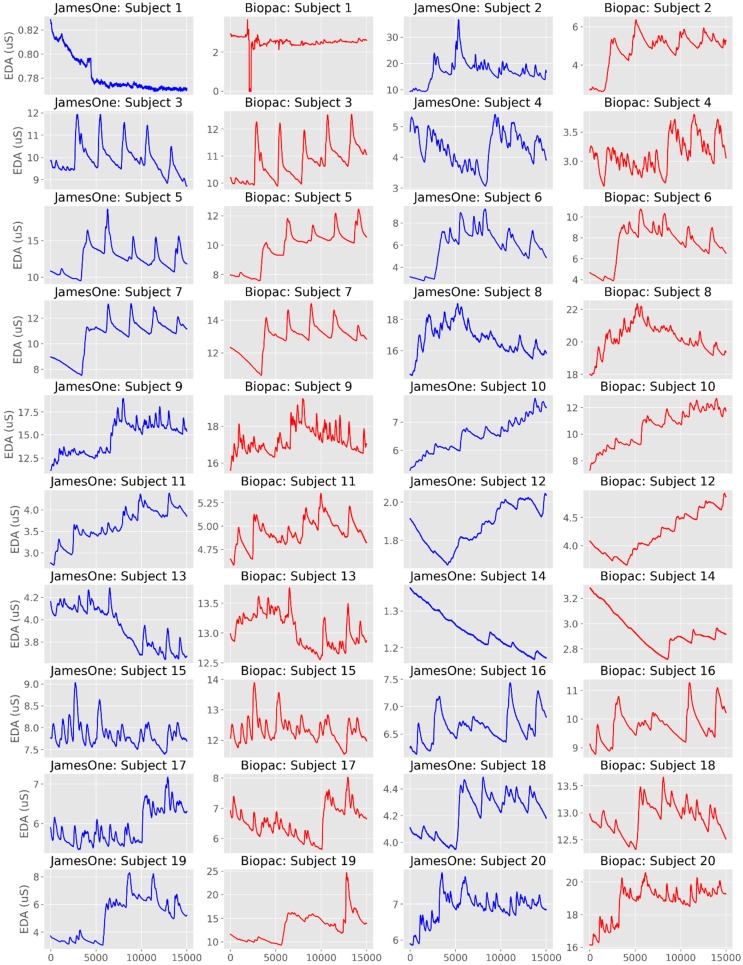
Individual time-series of the raw electrodermal activity (EDA) signals obtained with James One (blue lines) and Biopac (red lines) for Subjects 1–5 (**a**), 6–10 (**b**), 11–15 (**c**) and 16–20 (**d**). The x-axis represents time (in ms); the y-axis represents the EDA (in microsiemens).

**Figure 3:**
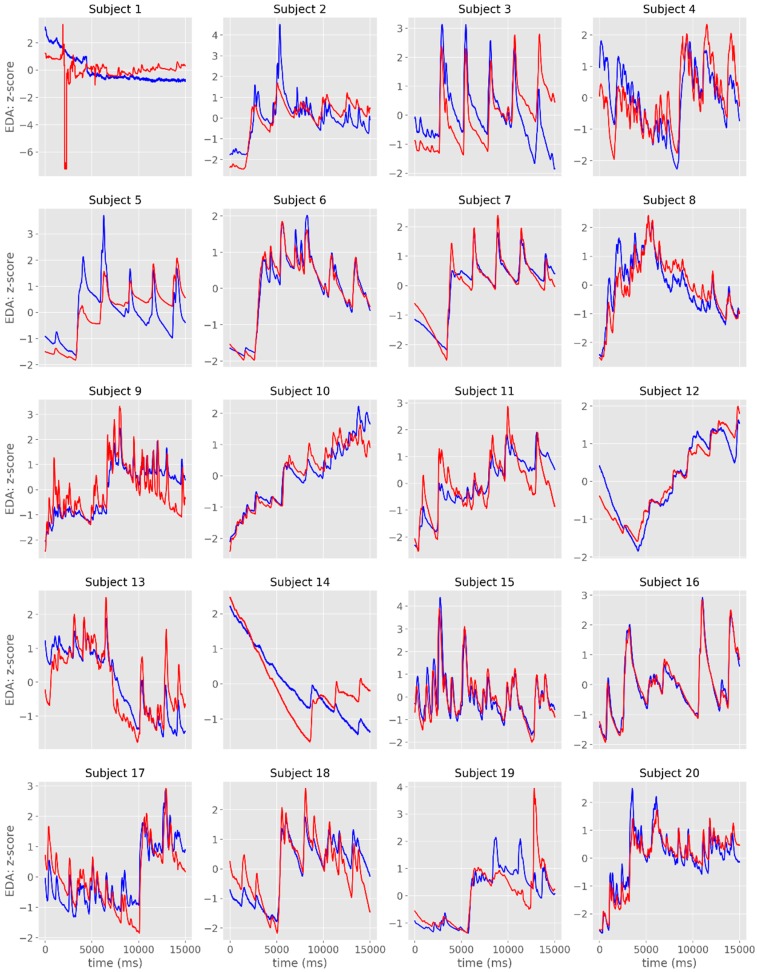
Individual time-series of the raw EDA signals obtained with James One (blue lines) and Biopac (red lines). The x-axis represents time (in ms); the y-axis represents the EDA (in standardized units).

**Figure 4:**
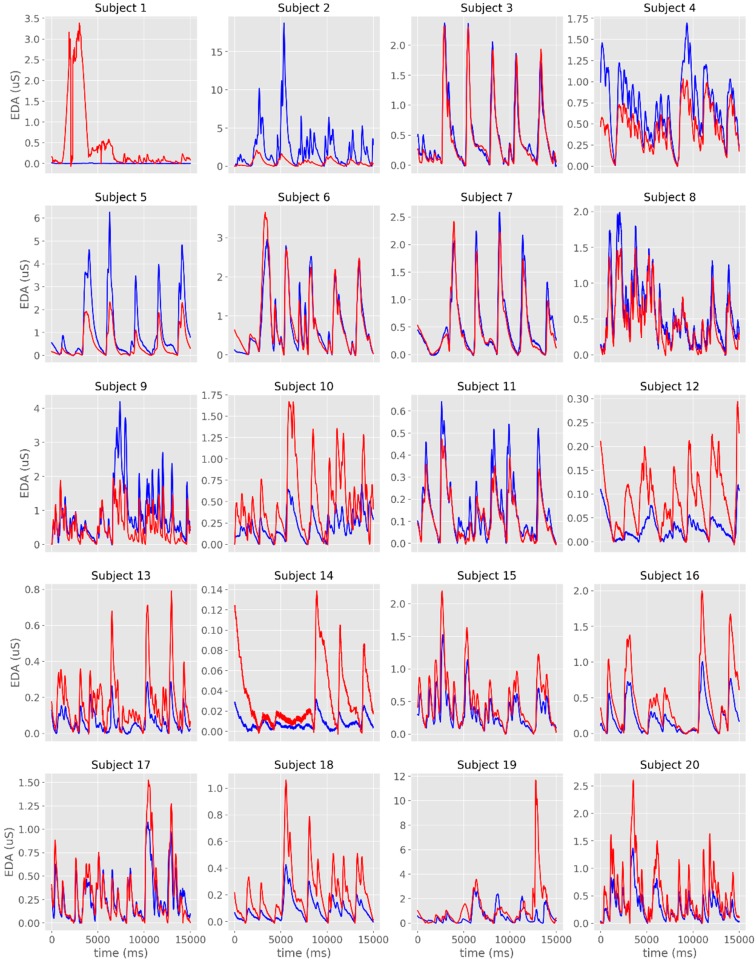
Individual time-series of the phasic component of EDA obtained with James One (blue lines) and Biopac (red lines). The x-axis represents time (in ms); the y-axis represents the EDA (in microsiemens).

The time-series from the raw EDA signals are plotted in Figure 2a,b and Figure 3. From the visual analysis of the plots, it is possible to observe that there is a considerable overlap between the two devices with regards to EDA assessment, with the exception of Subject #1, which had noisy records in both sensors. When excluding this participant from the different analyses, the average cross-correlation coefficients were: r = 0.839 for the raw signal [range: 0.501 (subject #3)–0.984 (subject #16)], r = 0.886 for the phasic signal in μS [range: 0.576 (subject #19)–0.973 (subject #3)], and r = 0.822 for the phasic signal in μS [range: 0.405 (subject #3)–0.991 (subject #6)] (Table 1, Figure 4). 

**Figure 5:**
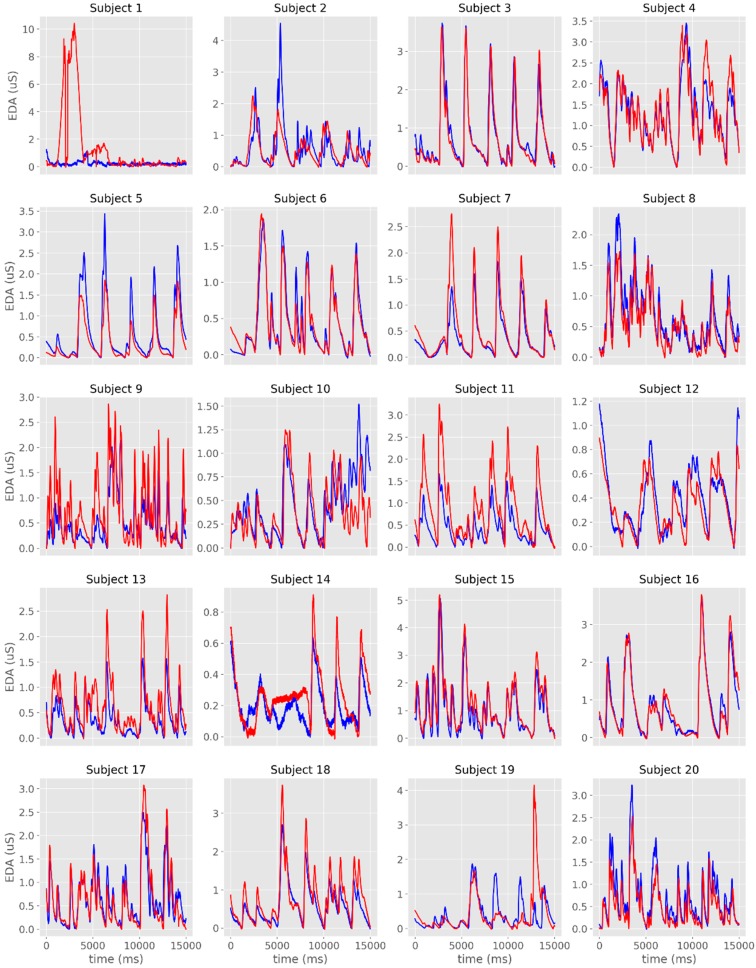
Individual time-series of the phasic component of EDA obtained with James One (blue lines) and Biopac (red lines). The x-axis represents time (in ms); the y-axis represents the EDA (in standardized units).

**Figure 6:**
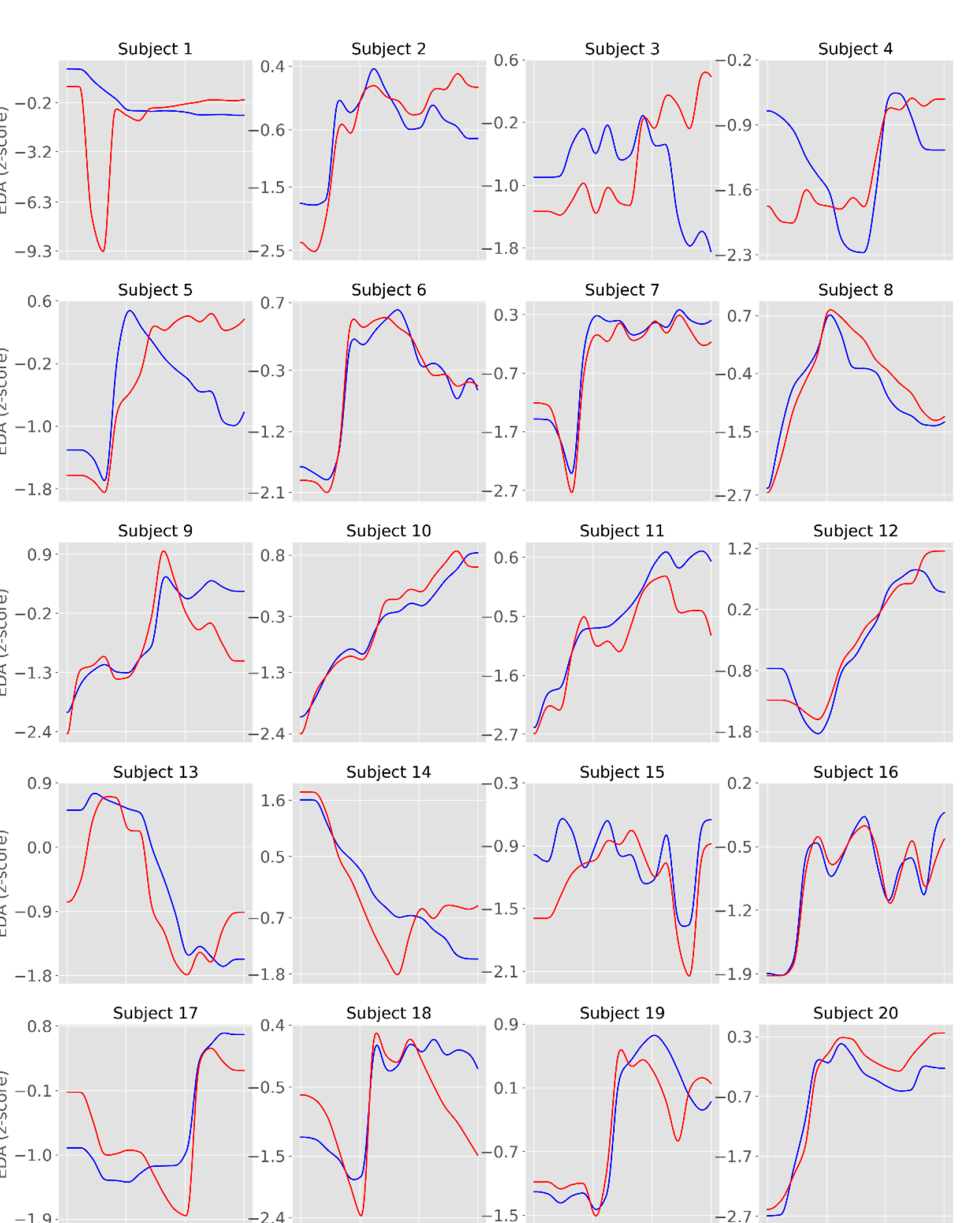
Individual time-series of the tonic component of the EDA signals obtained with the James One (blue lines) and the Biopac (red lines) devices. The x-axis represents time (in ms); the y-axis represents the EDA (in standardized units).

The overlap between signals was more evident when the time-series were displayed in standardized (z-scores) units (Figure 5). The visual representation of the tonic component revealed a less evident overlap between the signals recorded with the two systems, even though there were moderate to very large cross-correlations (Table 1, Figure 6).

**Figure 7:**
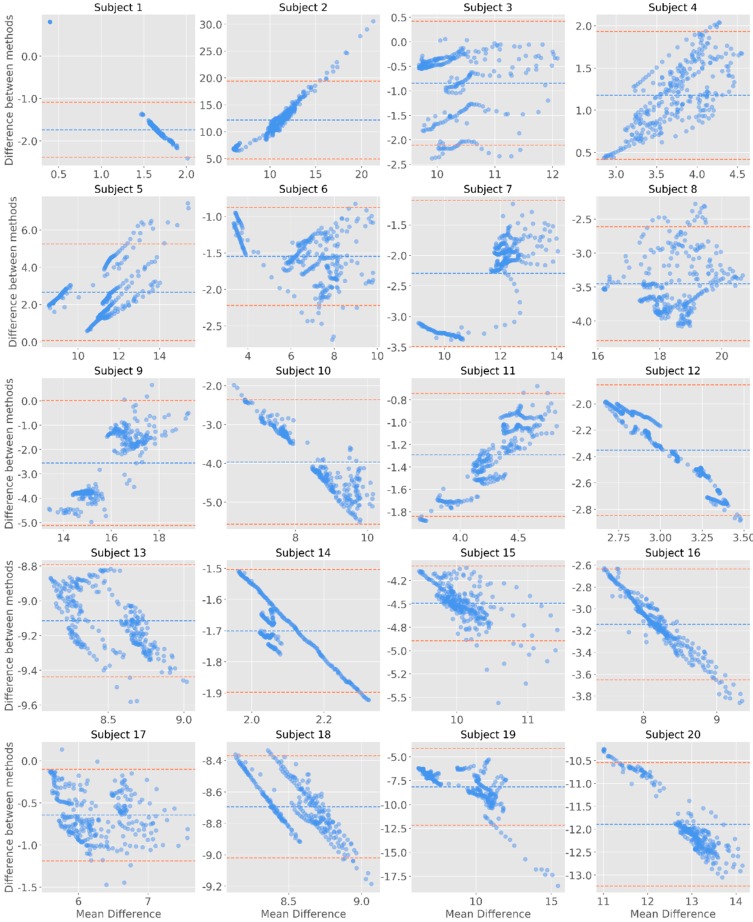
Bland-Altman plots for the raw signals. The y-axis represents the difference between the two signals (James One and Biopac); the x-axis represents the average of the two measures. The dashed blue line corresponds to the mean EDA value for each subject; the coral dashed lines are positioned 1.96 standard deviations away from the mean.

**Figure 8:**
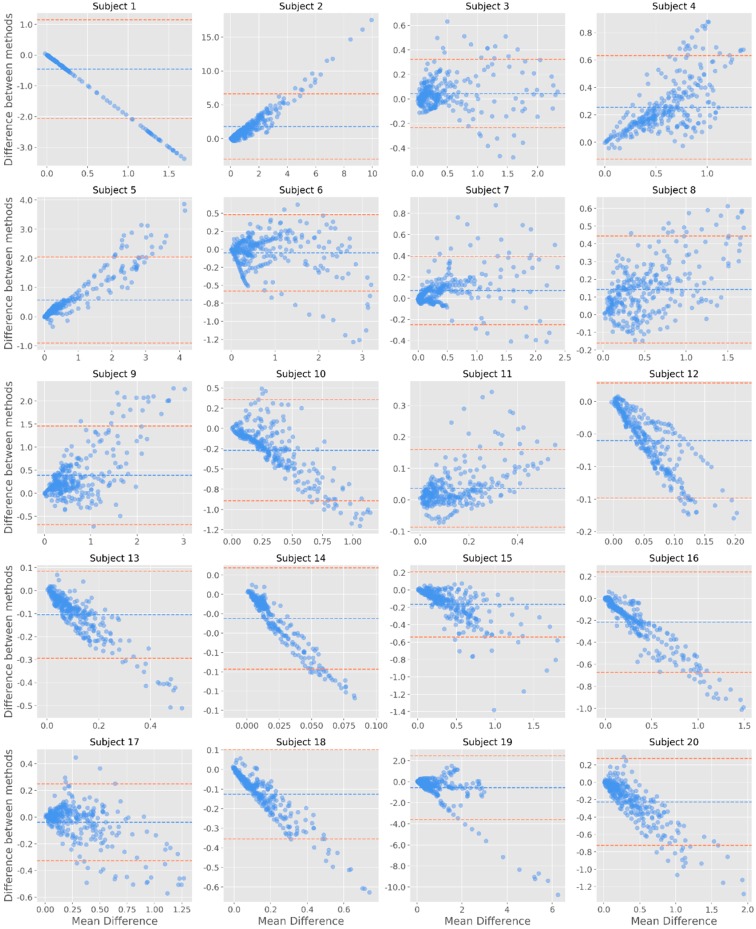
Bland-Altman plots for the phasic component of EDA. The y-axis represents the difference between the two signals (James One and Biopac); the x-axis represents the average of the two measures. The dashed blue line corresponds to the mean EDA value for each subject; the red dashed lines are positioned 1.96 standard deviations away from the mean.

**Figure 9:**
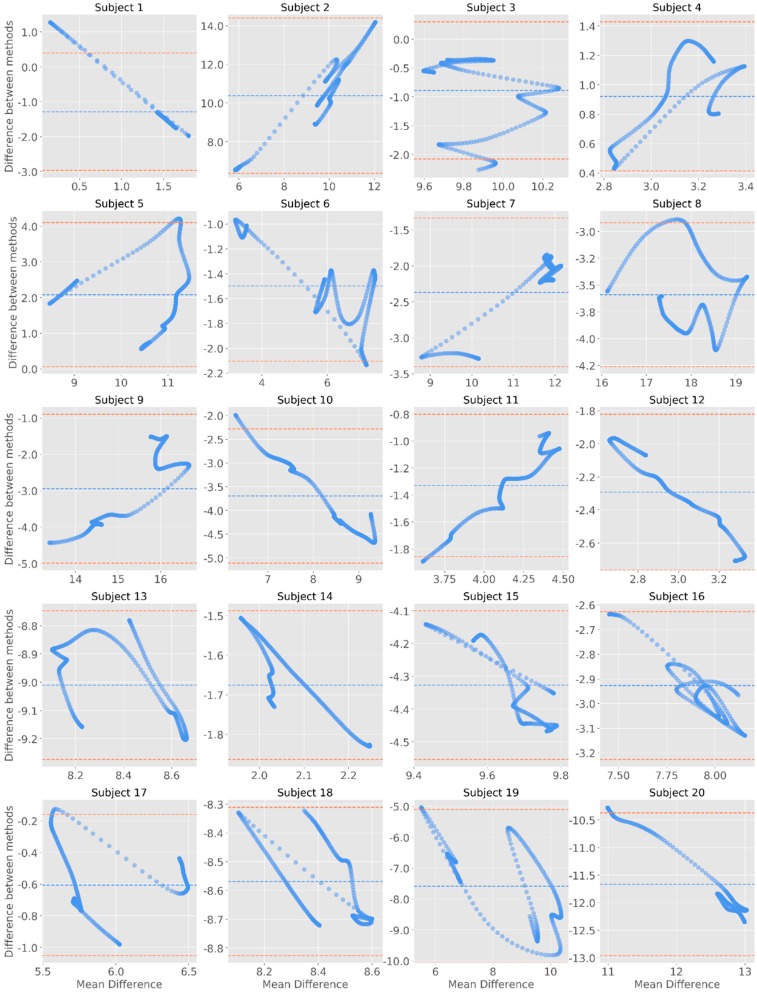
Bland-Altman plots for the tonic component of EDA. The y-axis represents the difference between the two signals (James One and Biopac); the x-axis represents the average of the two measures. The dashed blue line corresponds to the mean EDA value for each subject; the red dashed lines are positioned 1.96 standard deviations away from the mean.

The inspection of the Bland–Altman plots allows to observe that most datapoints are within the margin 95% confidence interval for the mean of differences (Figure 7, Figure 8 and Figure 9). For the raw signal, subjects #2, #3, #5, #8, #15, and #20 had the highest number of points falling outside the confidence interval—however representing less than 5% of the total number of datapoints (Figure 7). When considering the phasic component of EDA, it was noted that the differences were more pronounced for higher means. In fact, by comparing the Bland-Altman plots with the phasic signal (Figure 8), it can be noted that, in comparison to Biopac, James One recorded higher amplitudes for subjects #10, #12, #13, #14, #16, or #18. On the opposite, Biopac recorded higher amplitudes for subjects #4, #5, #9, and #11, which produced a positive slope on the Bland-Altman analysis of this component. 

Finally, regarding the tonic component of the signal, it was possible to note that less datapoints fall outside of the confidence interval—in this case, most studies do not have any datapoint falling outside of this margin (Figure 9). Considering these results, there does not seem to be evidence of systematic differences between the devices across the EDA signal.

### 3.2. Skin Conductance Responses

**Figure 10:**
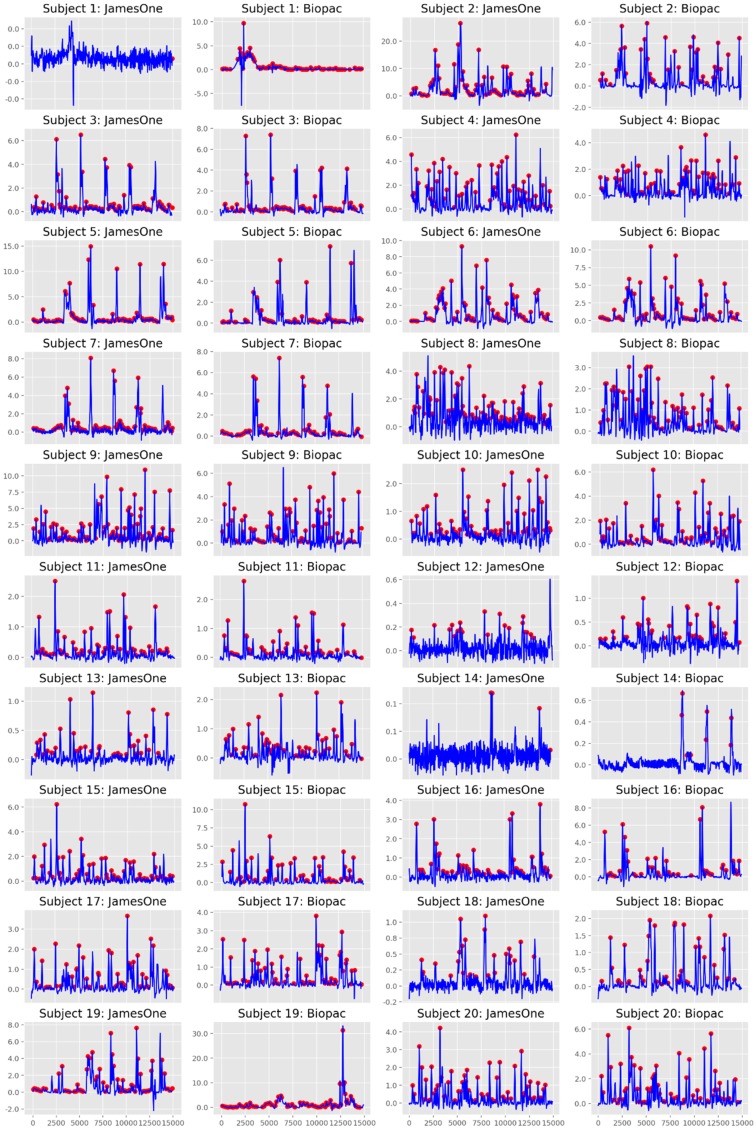
Identification of skin conductance responses (SCRs) for James One and Biopac. The blue lines represent the time-series of the phasic component of EDA, which are overlapped with the SCRs (red dots) identified at the threshold of 0.01 microsiemens.

**Figure 11:**
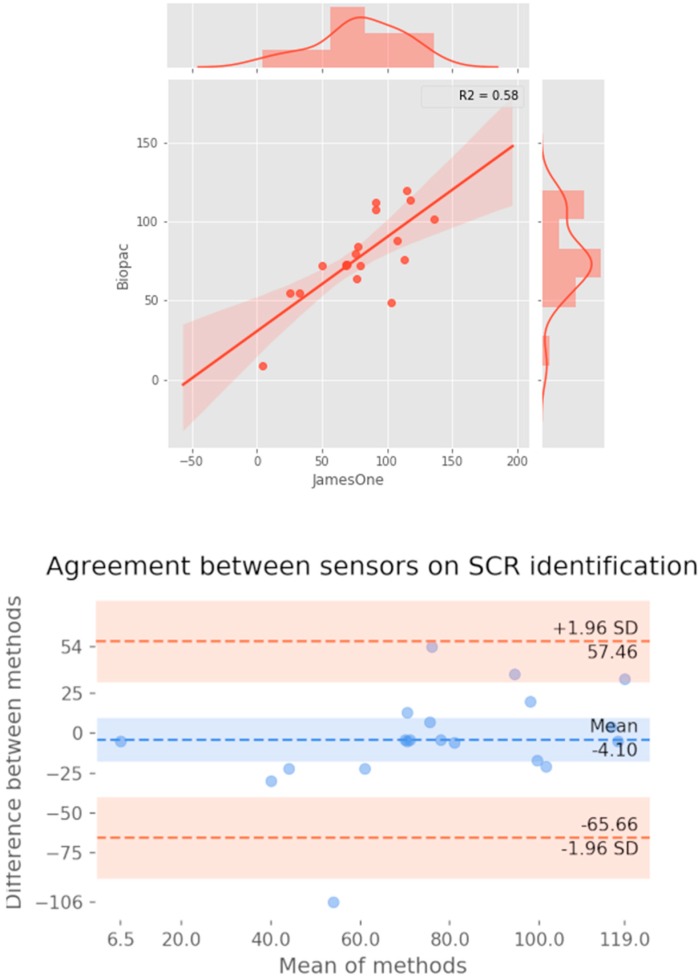
Association between the number of skin conductance responses identified at the threshold of 0.01 microsiemens between systems. **Top**: Scatter plot representing the magnitude of association; on the right and top ends of the plot, the histogram of the distribution of SCRs for each sensor is displayed. **Bottom**: Bland-Altman plot displaying the agreement between systems.

Regarding the quantification of SCRs (considering a threshold for detection of an SCR of 0.01 μS), it was noted that for most subjects, SCRs were similarly identified between the two devices (Figure 10). Across studies, the correlation coefficient had a strong magnitude (r = 0.760) (Figure 11). The Bland-Altman plot revealed that one of the points was outside the 95% confidence interval margin (Figure 11). Such result provides evidence for a satisfactory agreement between the sensors on the identification of SCRs.

### 3.3. Photoplethysmography

**Figure 12:**
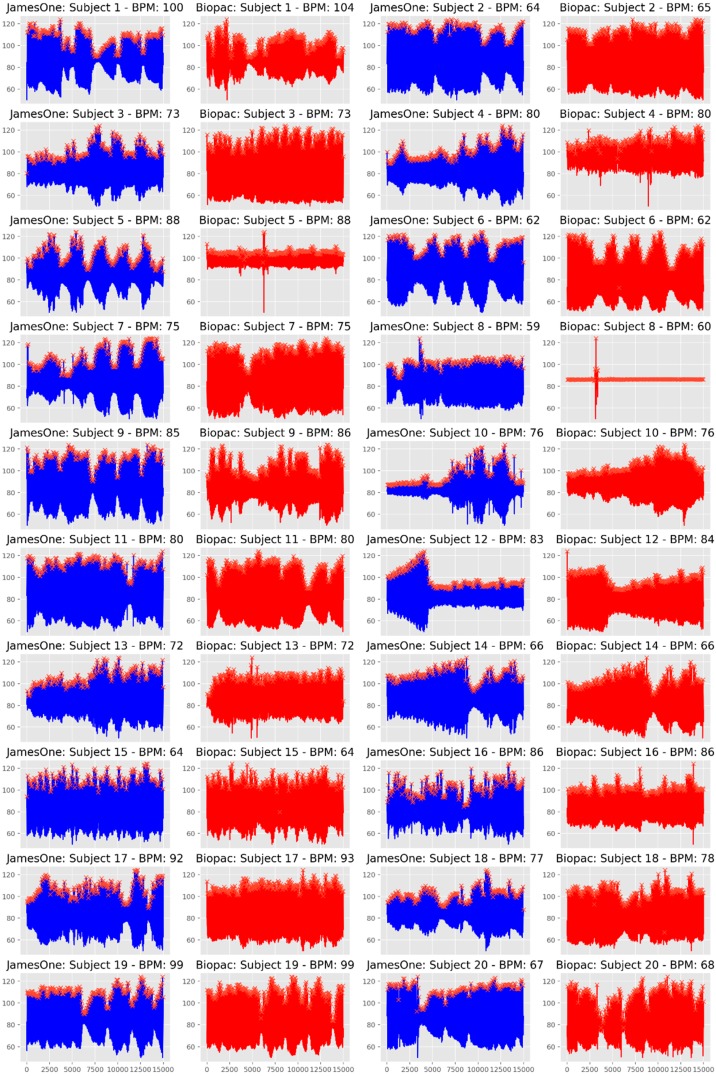
Whole time-series of the photoplethysmography (PPG) signal obtained with James One (**blue lines**) and Biopac (**red lines**).

**Figure 13:**
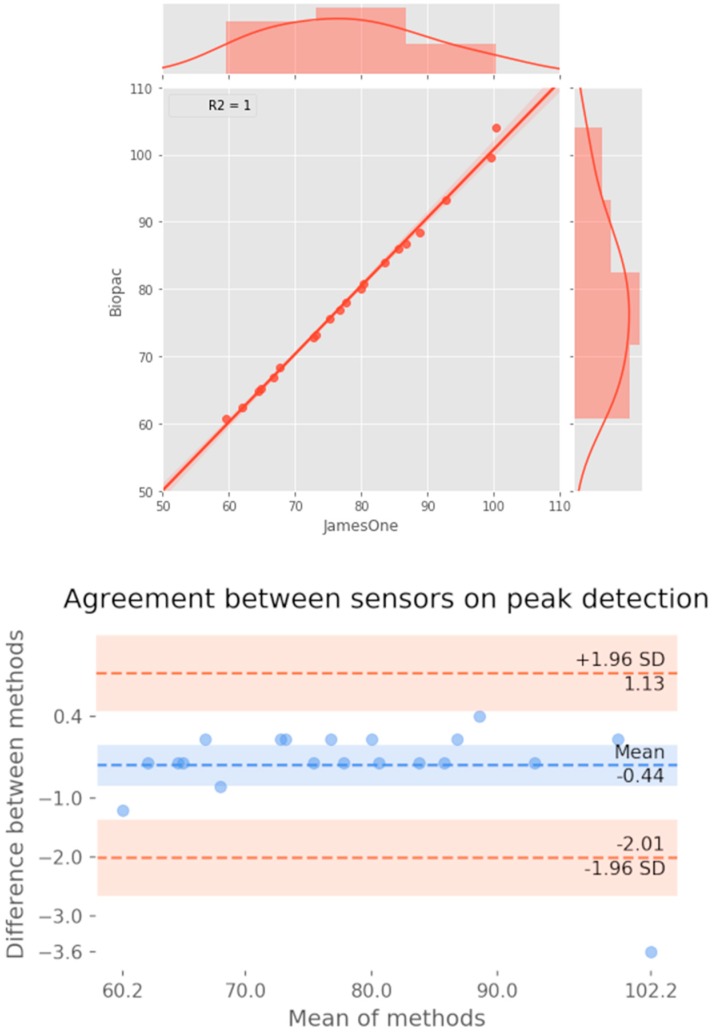
Association between the number of peaks detected for both systems. **Top**: Scatter plot representing the magnitude of association; on the right and top ends of the plot, the histogram of the distribution of SCRs for each sensor is displayed. **Bottom**: Bland–Altman plot displaying the agreement between systems.

The visual inspection of IBI time-series revealed that there were noisy signals for subjects #3, #5, #9, #17, #19. The grand-average for heart rate was 78.38 bpm (SD = 12.28) and 77.94 bpm (SD = 12.05) for James One and Biopac sensors, respectively. The visual inspection of the full signal demonstrates that there is a good convergence of the PPG signal between James One and Biopac (Figure 12). The average heart rate ranged from 59.6 (subject #8) to 100.4 bpm (subject #1) for the James One sensor and from 60.8 (subject #8) to 104.0 bpm (subject #1). The association between average HR for both signals had a large magnitude (r = 0.998), with an average difference of −0.440 bpm (SD = 0.838) and a maximum absolute deviation of 3.6 bpm (Table 2). However, this most pronounced difference was obtained for the subject with a noisy signal in both sensors. By excluding this participant from the analysis, the average difference decreased to −0.274 (SD = 0.340) and the correlation coefficient of the HR between devices increased to r > 0.999 (Figure 13). Also, the Bland–Altman plot evidenced that only one subject was outside of the 95% confidence interval of the difference between means. 

**Figure 14:**
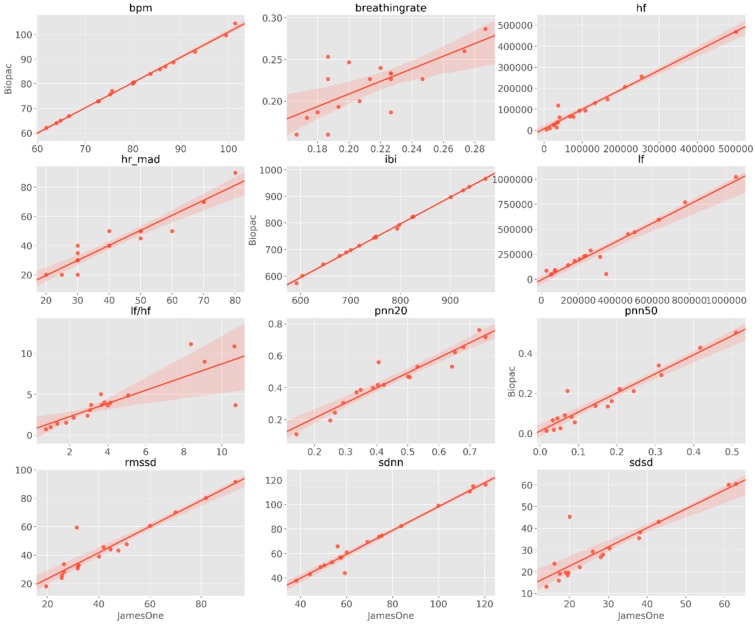
Scatter plots representing the magnitude of association across different metrics derived from the PPG signal. BPM—beats per minute, IBI—inter-beat interval, SDNN—standard deviation of RR intervals, SDSD—standard deviation of successive difference, RMSSD—root mean square of successive differences, pNN20—proportion of successive differences above 20 ms, pNN50—proportion of successive differences above 50 ms, MAD—median absolute deviation of RR intervals, LF-HRV—low-frequency HRV (range: 0.05–0.15 Hz), HF-HRV—high-frequency HRV (range: 0.15–0.5 Hz), HF/LF HRV—ratio between high-frequency and low-frequency HRV.

The correlations between HR/HRV metrics between the sensors are graphically represented in Figure 14 and statistically displayed in Table 3. Across the different metrics, the association with the lowest magnitude was obtained for BR (r = 0.712, *p* < 0.001); furthermore, except for the HF/LF ratio (r = 0.822, *p* < 0.001), all the remaining metrics had correlation coefficients higher than r = 0.900. Of relevance, the association between IBI had a correlation coefficient of r = 0.999. 

**Figure 15:**
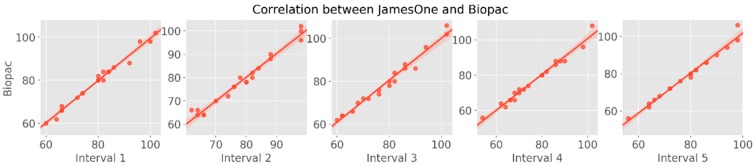
Scatter plots representing the magnitude of association of heart rate between devices: 30 s intervals of the PPG signal.

**Figure 16:**
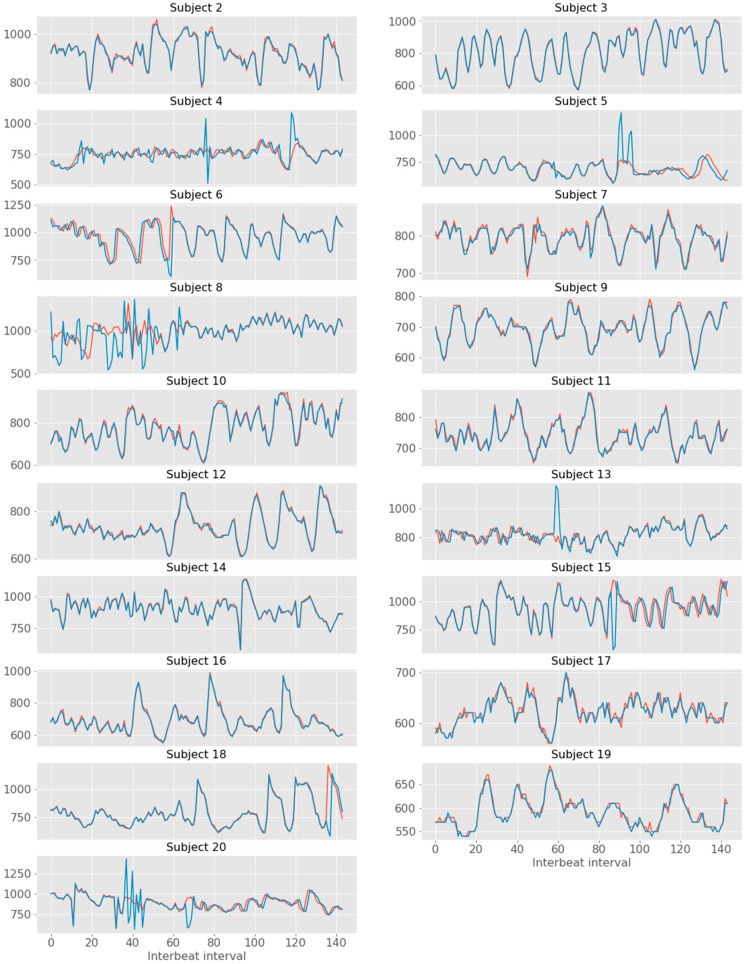
Time series of inter-beat intervals for James One (**red**) and Biopac (**blue**).

To further explore the dynamic association between the two devices with regards to the PPG signal, two complementary approaches were used: first, the similarity of PPG signal between the devices was analyzed in time-windows of 30 s; second, the distance between each HR peak was calculated to derive the time-series of the instantaneous IBI. For the first approach, we observed that the correlation coefficients between devices were very similar across intervals, with a range from r = 0.988 (3rd interval) to r = 0.992 (1st interval) (Figure 15). The cross-correlation between the two time-series demonstrated strong associations between James One and Biopac, ranging from r = 0.493 (subject #8) to r = 0.998 (subject #3) (Table 4, Figure 16).

## 4. Discussion

In this work, we aimed to test the validity of the psychophysiological signals obtained with the James One device, as compared with a gold-standard system for studies within the psychophysiological field. We observed that the EDA and PPG signals measured with the James One device display strong associations with the Biopac system.

The cross-correlation coefficients indicated a strong association between the James One and Biopac systems, considering the raw signals. Furthermore, when decomposing the EDA signal into phasic and tonic components, by means of a continuous decomposition analysis, the same pattern was observed. Here, even though there seems to be a reduced overlap between the oscillations of the time-series of the tonic activity, the signals present a high degree of covariation between each other, as demonstrated by the high magnitude of the cross-correlation coefficients. In addition to this, the graphic visualization of Bland-Altman plots provided evidence that James One captures the dynamics of EDA at a comparable fashion to that of Biopac—a laboratory-grade research equipment (few datapoints were outside of the 95% confidence interval for the difference between means), considering the raw and the phasic and tonic components of the signal. Furthermore, there was a strong association between the number of skin conductance responses across the devices. With regards to the PPG signal, we observed that peaks were very similarly identified in both James One and Biopac—with an almost perfect linear correlation (R^2^ > 0.999). Of note, very high levels of association were preserved after a robustness analysis, in which the correlation between the number of peaks was performed in time-intervals of 30 s. Similarly, there were strong associations between metrics of HRV, including time-domain metrics (e.g., IBI, RMSSD) and frequency-domain metrics (HF, LF, and HF/LF ratio). In the case of IBI, the correspondence between signals was performed at a dynamic level, in which we observed high cross-correlation coefficients on the IBI time-series.

Of note, it is relevant to highlight that such comparable results were obtained, even though the acquisition methods were different across devices: (1) The sensors were placed on different hands (left hand for Biopac, right for James One)—which could lead to systematic differences related with laterality effects; (2) the fixation method for EDA is different between systems (the Biopac system uses metallic clamps, while James One fixates directly onto the electrodes via a metallic fast snap); and (3) while James One’s EDA sensor is placed on the palm of the hand (where there is an increased density of eccrine sweat glands [22]), for Biopac, the EDA electrodes are placed on fingers. With this in mind, it seems reasonable to advocate that the James One sensor is capable of robustly collecting psychophysiological data, while having the advantage of requiring considerably lower setup times in comparison with the laboratorial equipment. This enables a scalable use of peripheral psychophysiological monitoring with the potential of reducing the barriers between rigorous laboratory-based data collections and the ecological validity for such signals to be acquired in realistic environments, where the participants can be assessed in real-time, in their naturalistic context. Also, the portability and low-cost of the James One system potentiates the use or larger sample sizes to face the persistent problem of lack of statistical power in scientific experiments. For instance, a recent meta-analytic investigation concluded that the median sample sizes for comparing groups in HRV experiments are 23 and 24, for control and experimental groups, respectively—and that such sample dimensions are not sufficiently powered to detect small or medium effect sizes [23]. Thus, the use of sensors that can capture remotely reliable psychophysiological signals can constitute a contribution of upmost relevance to the field, enabling the use of more powered studies, and consequently contributing to face the reproducibility crisis in psychological [24] and biomedical research [25]. 

Notwithstanding, it is also important to reinforce that this study was performed in standard laboratorial conditions with a controlled data acquisition pipeline. While we considered that this would be the first step to demonstrate the comparability of the James One with one of the systems most widely used in peripheral psychophysiological acquisitions—Biopac—it will be relevant to further assess the potential of James One to be used in ecological contexts, outside of a laboratorial setting. We foresee the potential benefits of such approach in multiple domains. Decades of scientific research have enabled a comprehensive characterization of a variety of clinical conditions and psychological phenomena with regards to their peripheral psychophysiological correlates. Measures of the autonomic nervous system (ANS) are robustly associated with emotional processing [26]. The variation of EDA metrics are directly associated with self-reported arousal [27]. A recent review of the literature has demonstrated that mood, anxiety, psychosis, and dependent disorders are associated with consistent reductions of HRV [28]. Furthermore, ANS measures are being increasingly used for the characterization of psychological states, such as stress detection [29,30,31]. In fact, the ANS seems to play an important role in the susceptibility to stress and thus ANS manipulation with biofeedback techniques has been proposed to promote resilience [32]. In addition, it has been proposed that the use of biofeedback techniques can play a potential therapeutic effect in conditions such as epilepsy or Tourette syndrome [33]. Impaired emotional regulation is a core feature of distinct psychopathological conditions, with an important impact on human development [34]. Biofeedback-based interventions have also been associated with improvements related with depression, anxiety, or attention in elderly individuals [35]. Altogether, it has becoming progressively accepted that interventions tackling the self-modulation of psychophysiological signals may be used to complement “standard” psychotherapeutic approaches. These techniques have the potential of providing non-invasive psychophysiological interventions with a beneficial impact on different psychological conditions [36]. 

While for the purpose of this research, we were essentially interested in the characterization of the similarity between the James One and Biopac, we did not approach the modulation of psychological processes or the characterization of clinical populations, we clearly consider that this is a path to be tackled in future investigations. Given the abovementioned involvement of ANS in health-related processes, a portable psychophysiological system can be useful for an ecological assessment of these measures with the purpose of their monitoring and/or self-modulation. Having this in mind, in the future we will be interested in assessing the feasibility of the James One device with such application.

These results illustrate the feasibility of using a small, wireless, and unobtrusive device for EDA data collection. Because of the ease associated with the preparation of recordings, the use of this type of equipment may bring additional value to psychophysiological experiments, by allowing the collection of robust signals with reduced setup times and non-invasive record sessions. As such, the James One system may constitute an optimal solution for autonomous data collection, allowing self-recordings with minimal training—which may potentiate the collection of psychophysiological data at a larger scale.

## Figures and Tables

**Table 1:** Individual cross-correlation values for the raw and phasic electrodermal activity.

Subject	Raw	Phasic (μS)	Phasic (z-Score)	Tonic (μS)	Tonic (z-Score)
1	0.157829	0.566533	0.507982	0.204574	0.203871
2	0.777349	0.751913	0.777177	0.851708	0.858796
3	0.501085	0.973458	0.971107	0.404898	0.396931
4	0.743937	0.875577	0.900557	0.464637	0.492426
5	0.689561	0.957669	0.956994	0.594456	0.606494
6	0.983215	0.951585	0.957592	0.990673	0.985216
7	0.959799	0.961409	0.946377	0.979678	0.976061
8	0.893347	0.950241	0.944622	0.897131	0.918484
9	0.75305	0.78268	0.775502	0.755483	0.742987
10	0.936485	0.878783	0.747058	0.968315	0.980932
11	0.823657	0.951024	0.948961	0.928863	0.925523
12	0.946632	0.772801	0.852358	0.956802	0.952326
13	0.832333	0.894016	0.892842	0.872568	0.864734
14	0.80463	0.820812	0.818961	0.808144	0.797832
15	0.943365	0.970063	0.958886	0.801582	0.650859
16	0.984161	0.972005	0.971048	0.973049	0.969597
17	0.83786	0.907575	0.901858	0.801852	0.799356
18	0.852281	0.953636	0.926891	0.715306	0.69144
19	0.742936	0.576188	0.56903	0.86492	0.911805
20	0.927842	0.939918	0.939204	0.981458	0.965082

**Table 2:** Individual heart rate (in bpm) for the Biopac and James One.

Subject	BP	James One	Diff
1	104	100.4	3.6
2	65.2	64.8	0.4
3	73.2	73.2	0
4	80	80	0
5	88.4	88.8	0.4
6	62.4	62	0.4
7	75.6	75.2	0.4
8	60.8	59.6	1.2
9	86	85.6	0.4
10	76.8	76.8	0
11	80.8	80.4	0.4
12	84	83.6	0.4
13	72.8	72.8	0
14	66.8	66.8	0
15	64.8	64.4	0.4
16	86.8	86.8	0
17	93.2	92.8	0.4
18	78	77.6	0.4
19	99.6	99.6	0
20	68.4	67.6	0.8
Average	78.38	77.94	0.48
SD	12.28248	12.05217	0.813914
r	0.997839184		

**Table 3:** Association between heart rate variability (HRV) metrics for the James One and Biopac devices.

Metric	r	P
Beats per minute (BPM)	0.998412	2.02 × 10^−21^
Breathing rate (BR)	0.711613	9.26 × 10^−4^
High-frequency HRV (HF-HRV)	0.982407	4.37 × 10^−13^
Median absolute deviation of RR intervals (MAD)	0.951843	1.25 × 10^−9^
Inter-beat Interval (IBI)	0.999173	1.10 × 10^−23^
Low-frequency HRV (HF-HRV)	0.96339	1.45 × 10^−10^
Ration between high-frequency and low-frequency HRV (HF/LF-HRV)	0.822172	2.83 × 10^−5^
Proportion of successive differences above 20 ms (pNN20)	0.95373	9.13 × 10^−10^
Proportion of successive differences above 50 ms (pNN50)	0.95697	5.16 × 10^−10^
Root mean square of successive differences (RMSSD)	0.940546	6.51 × 10^−9^
Standard deviation of RR intervals (SDNN)	0.984705	1.44 × 10^−13^
Standard deviation of successive differences (SDSD)	0.901866	3.17 × 10^−7^

**Table 4:** Cross-correlation coefficient between the including interbeat interval (IBI) time-series for each participant.

Subject	IBI
2	0.991393
3	0.997535
4	0.585372
5	0.710497
6	0.821138
7	0.95914
8	0.492672
9	0.987846
10	0.986796
11	0.983388
12	0.991468
13	0.673156
14	0.991269
15	0.771187
16	0.993361
17	0.958586
18	0.854538
19	0.982767
20	0.62377
